# A computational approach for deciphering the interactions between proximal and distal gene regulators in GC B-cell response

**DOI:** 10.1093/nargab/lqae050

**Published:** 2024-05-06

**Authors:** Sung-Joon Park, Kenta Nakai

**Affiliations:** Human Genome Center, The Institute of Medical Science, The University of Tokyo, 4-6-1 Shirokanedai, Minato-ku, Tokyo 108-8639, Japan; Human Genome Center, The Institute of Medical Science, The University of Tokyo, 4-6-1 Shirokanedai, Minato-ku, Tokyo 108-8639, Japan

## Abstract

Delineating the intricate interplay between promoter-proximal and -distal regulators is crucial for understanding the function of transcriptional mediator complexes implicated in the regulation of gene expression. The present study aimed to develop a computational method for accurately modeling the spatial proximal and distal regulatory interactions. Our method combined regression-based models to identify key regulators through gene expression prediction and a graph-embedding approach to detect coregulated genes. This approach enabled a detailed investigation of the gene regulatory mechanisms for germinal center B cells, accompanied by dramatic rearrangements of the genome structure. We found that while the promoter-proximal regulatory elements were the principal regulators of gene expression, the distal regulators fine-tuned transcription. Moreover, our approach unveiled the presence of modular regulators, such as cofactors and proximal/distal transcription factors, which were co-expressed with their target genes. Some of these modules exhibited abnormal expression patterns in lymphoma. These findings suggest that the dysregulation of interactions between transcriptional and architectural factors is associated with chromatin reorganization failure, which may increase the risk of malignancy. Therefore, our computational approach helps decipher the transcriptional *cis*-regulatory code spatially interacting.

## Introduction

Genes of multicellular organisms are regulated via a diverse set of mechanisms, which are often cell type specific. Growing evidence supports the notion that the dynamic rearrangement of the spatial genome architecture affects the interactions among *cis*-regulatory elements in the nuclear microenvironment, constituting the so-called transcriptional domain. Within this domain, distal regulatory elements (e.g. enhancers, silencers and insulators) communicate with promoter-proximal regulatory elements and exhibit modularity, cooperativity and specificity in spatiotemporal control of gene expression (1–3). These interactions together mediate gene regulation in three-dimensional (3D) space. Therefore, deciphering the *cis*-regulatory code in the 3D transcriptional domain is fundamentally important for understanding the diverse roles and mechanisms of gene regulation.

Over the years, many studies have investigated the role of the interactions among promoter-proximal and -distal regulatory elements in processes such as cell fate decisions and disease development (4,5). For instance, dramatic genome rearrangement occurs during the transition from naive B (NB) cells to germinal center B (GCB) cells. This rearrangement involves many locus control regions and enhancers, which affect the expression of thousands of cell-type-specific genes (6–8). However, our understanding of the roles of enhancers and associated signaling transfer during promoter targeting is still very limited. One of the primary challenges is the sheer complexity of the gene regulatory networks (6,9), which are composed of *cis*-regulatory elements interacting with multiple transcription factors (TFs) and architectural proteins (5,10,11). Although recent computational approaches have made considerable breakthroughs in characterizing the genome sequences of important regulatory elements (12,13), there has been less focus on dissecting their multiway interactions.

The aim of the present study was therefore to use a computational method to model the interactions among promoter-distal and -proximal transcriptional regulators and associated cofactors, collectively referred to as the ‘3D regulatory interaction’ of gene expression. After defining the 3D transcriptional domains using Hi-C (high-throughput chromosome conformation capture) contact information, we profiled TF binding sites (TFBSs) identified in the domains. Then, we inferred key factors from the profiles by applying a regression-based algorithm (14). A graph-embedding algorithm (15), which characterized cofactor–TF–gene networks, was subsequently used to detect coregulated sets of cell-type-specific genes and their regulators. By applying this novel approach to the investigation of the regulation of human GC B-cell response, we discovered that the interplay between TFs and architectural factors is essential for maintaining the function of 3D gene regulatory networks within the transcriptional domains. Our findings highlight the importance of understanding the dynamics of spatial regulatory interactions that could help improve the efficiency of therapeutic biomarker discovery.

## Materials and methods

### Data preparation

The raw and processed NB and GCB datasets were downloaded from the Gene Expression Omnibus (GEO), under accession numbers GSE84022 (for RNA-seq FASTQ files), GSE159314 (for ATAC-seq narrow peaks) and GSE84022 (for Hi-C contacts). Data relating to the narrow peaks of the six histone marks H3K4me1, H3K4me3, H3K9me3, H3K27me3, H3K36me3 and H3K27ac were downloaded from DeepBlue (16). The RNA-seq FASTQ files generated using the following cancer cell lines were obtained from the Cancer Cell Line Encyclopedia (17): OCI-LY10 (Ly10), activated B-cell-like (ABC) subtype of diffuse large B-cell lymphoma (DLBCL); OCI-LY7 (Ly7), GCB-cell subtype of DLBCL; BL-41, Burkitt lymphoma; and Mino, mantle cell lymphoma. Data relating to the ChIP-seq peaks of *BACH2* (GSM1084800), *MAFK* (GSM1159670) and *MYC* (GSM762710) were also downloaded from GEO.

The enhancer–promoter interactions (EPIs) screened by the CRISPRi-FlowFISH method for chronic myeloid leukemia (CML) cell line K562 were downloaded from the original study (11). The EPIs predicted by the ABC model (11) for acute monocytic leukemia (AML) cell line THP-1 and OCI-LY7 were downloaded from the published research (18). The gene expression profiles for K562 and THP-1 were downloaded from DepMap (https://depmap.org/portal/), and the histone modifications and chromatin accessibility datasets were prepared from ENCODE, BLUEPRINT and GEO, as summarized in Supplementary Table S1.

### Data processing

After assessing the RNA-seq reads using Trimmomatic (version 0.39) with the option ‘ILLUMINACLIP:adapter_file:2:30:10 LEADING:20 TRAILING:20 MINLEN:36’ (19), HISAT2 (version 2.2.1) (20) was used to align the quality-controlled reads to the hg38 human reference genome. Cufflinks (version 2.2.1), with RefSeq coding-gene annotation (21), was used to quantify gene expression levels in terms of fragments per kilobase of exon per million reads mapped (FPKM) by merging multiple replicates. All the FPKM values were then normalized to transcripts per million (TPM) values. Of note, any genes that included RefSeq noncoding IDs (e.g. *IRF4*) were removed to avoid ambiguity in expression quantification. The peak positions of the ChIP-seq and ATAC-seq datasets were retrieved using *q*-value <0.05 and a >2 fold change (FC); regions that did not overlap with the blacklisted genomic regions were retained (22).

The contact frequencies of intrachromosomal Hi-C anchor pairs were normalized by the Vanilla-Coverage approach (23). When the center of an anchor located at intergenic regions connected with its partner anchor situated >10 kb away and at a core promoter, spanning −2500 to +500 bp from a transcription start site (TSS), the center of that intergenic anchor was bidirectionally stretched to a 5-kb window and defined as a long-range contact (LRC) for a corresponding gene.

### Scoring TFBSs in the promoter region

MATCH in the minimize false-positive mode (24) with TRANSFAC DB (https://www.genexplain.jp/transfac.html) was used to scan for TFBSs from the DNA sequences of the proximal promoter region (−10 kb from the TSS). It is noteworthy that this DB did not include the TFBSs of *IRF8* and *FOXO1*, which is known to control GC B-cell response. Considering that a TFBS may be detected repeatedly from a promoter region and bound by multiple TFs during gene expression, a TFBS *j* that was found *K* times within the promoter *i* and potentially targeted by *N* number of TFs was scored as follows:


\begin{equation*}{\rm TFBS}\_{{s}_{ij}} = \mathop \sum \limits_{k = 1}^K \left( {{{L}_{ik}} \times \mathop \sum \limits_{n = 1}^N {{{\log }}_{10}}\,{{{\rm TPM}}_{jn}}} \right),\end{equation*}

where *L_ik_* represents a weight based on the location of the *k*th TFBS*_j_* in the promoter *i* and TPM*_jn_* is the expression level of TF*_n_* that binds to the TFBS. As in previous studies (14,25), the weight *L_ik_* was defined using histograms displaying ${{D}_{{\rm obs}}}$, which represents the distribution of TFBS*_j_* positions observed across all the promoters, and ${{D}_{{\rm rnd}}}$, which represents the random distribution of TFBS*_j_*, calculated as follows:


\begin{equation*}{{L}_{ik}} = \left\{ {\begin{array}{@{}*{2}{l}@{}} 0,&\quad{{\rm if}\; {{D}_{{\rm obs}}}\left( b \right) \le {{D}_{{\rm rnd}}}\left( b \right),}\\ {\frac{{{{D}_{{\rm obs}}}\left( b \right) - {{D}_{{\rm rnd}}}\left( b \right)}}{{{{D}_{{\rm obs}}}\left( b \right)}}},&\quad{{\rm if}\; {{D}_{{\rm obs}}}\left( b \right) >{{D}_{{\rm rnd}}}\left( b \right),} \end{array}} \right.\end{equation*}

where *b* corresponds to the index of 100-bp bins in which the location of the *k*th TFBS*_j_* is contained.

### Scoring TFBSs in the LRC region

To detect TFBSs from LRC regions (i.e. lrcTFBSs), the DNA sequences of LRC regions were scanned by the same procedure for dealing with promoter sequences. Considering that a promoter may contact multiple 5-kb LRCs, lrcTFBSs may appear multiple times within the same LRC regions or originate from different LRC regions. Thus, an lrcTFBS *j* found *K* times from the *C* number of LRCs contacted to the core promoter *i* was scored as follows:


\begin{equation*}{\rm lrcTFBS}\_{{s}_{ij}} = \mathop \sum \limits_{c = 1}^C \mathop \sum \limits_{k = 1}^K \left( {{{A}_{ic}} \times {{M}_{ick}} \times \mathop \sum \limits_{n = 1}^N {{{\log }}_{10}}\,{{{\rm TPM}}_{jn}}} \right),\end{equation*}

where *A_ic_* stands for the activity of the *c*th LRC region for the promoter *i* and TPM*_jn_* is the expression level of TF*_n_* binding to lrcTFBS*_j_*. *M_ick_* represents the MATCH score (24), which is a score, ranging from 0.7 to 1.0, used to grade the alignment of lrcTFBS*_j_* with the LRC sequences. The activity *A_ic_* was calculated as


\begin{equation*}{{A}_{ic}} = \frac{{{{{\rm HM}}_{ic}} \times {{{\rm CT}}_{ic}}}}{{\mathop \sum \nolimits_{m = 1}^T {{{\rm HM}}_{im}} \times {{{\rm CT}}_{im}}}},\end{equation*}

where *T* represents the total number of distinct LRC regions contacted to the promoter *i*, and HM and CT stand for the fold-enrichment (FE) value of histone modification and the normalized Hi-C contacts, respectively. The histone FE is the mean of the log_2_ FE signals of H3K27ac and H3K4me1 peaks found within the 5-kb LRC regions. Note that we employed the scoring equation in the activity-by-contact model (11) to approximate the activity *A_ic_*, a relative effect of an LRC region on the target gene expression. The lrcTFBSs are weighted by the corresponding LRC activity *A* incorporated with the signals of the enhancer-specific histone marks.

### Building regression models

Given a set of TPM values as response variables, a linear regression model predicts the TPM value with *n* number of explanatory variables (i.e. regulatory features) by estimating regression coefficients (RCs) as follows:


\begin{equation*}{{\log }_{10}}\,{{{\rm TPM}}_i} = \mathop \sum \limits_{j = 1}^n {{w}_j}{{S}_{ij}} + {{e}_{i }},\end{equation*}

where *S_ij_*_and_*w_j_* represent a regulatory feature *j* for the gene *i* and an RC for the feature *j*, respectively. The positive RC implies the regulatory effect as an activator, while the negative RC stands for repressive effect. Conducting comparative studies using previously published models is challenging and may lead to biased interpretations. To address this issue, new models were constructed specifically in the present study. These models used four distinct sets of regulatory features, each of which had a different number of explanatory variables: (i) the baseline model (B model) using TFBS_*s_ij_* within 10-kb TSS upstream regions; (ii) the BH model was an extended version of the B model, which also included the log_2_ FEs of each of the six histone markers located in the promoter regions; (iii) the BL model was an extended version of the B model, which was generated by additionally incorporating lrcTFBS_*s_ij_*; and (iv) the BHL model was constructed by combining the BL and BH models. Randomized versions of the above models were also generated.

### Feature selection procedure

An iterative procedure was used to find statistically significant explanatory variables through a greedy search (Supplementary Figure S1) as previously described (14). In brief, stepwise model selection, based on Akaike’s information criterion (AIC), was used to predict the most adequate explanatory variables from the full regression model. This process yielded the reduced model M1, the predictive power of which was evaluated using 5-fold cross-validation (CV). Next, the M1 was extended by introducing a variable randomly selected from the set of variables that AIC had removed in the previous step; this process generated the trial model M2. The performance of M2 was assessed using AIC and 5-fold CV. If M2 demonstrated improved predictive power over M1, it was selected; otherwise, another M2, with a different random variable, was subsequently tested. This procedure was repeated until all the variables removed during the AIC step had been tested. Each iteration of this feature selection procedure was performed 10 times by specifying different random seeds, which yields an ensemble of RCs.

### Graph embedding

Using significant TFBSs identified by the regression model, a network of TF–gene interactions was constructed and subsequently extended to form a network of cofactor–TF–gene interactions (Supplementary Figure S1). The cofactors were prepared from STRING (version 12.0) (26) as physically interacting proteins with any of the TFs: the experimentally validated physical protein–protein interactions (PPIs) were retrieved with a confidence score (PPI score) >0.4. The TF- and cofactor-coding genes expressed (>3 TPM at NB and/or GCB cells) were used to build the network to be embedded. In graph-embedding methods, specifically the large-scale information network embedding (LINE) model (15,27), each vertex in the network (i.e. TF-coding genes, cofactor-coding genes or cell-type-specific genes in this study) was represented by a 200-dimensional vector based on the similarity of subgraph structures. For instance, in a binary-weighted undirected graph, the one-step neighbor of a vertex (i.e. a subgraph structure with the first-order proximity) and the neighbor’s neighbor of the vertex (i.e. a subgraph structure with the second-order proximity) are preserved in a low-dimensional dense vector space. To optimize the embedding vectors, an asynchronous stochastic gradient algorithm with negative edge sampling was used to minimize the two objective functions of the first- (*O*_1_) and second-order (*O*_2_) proximities, based on the KL divergence as a distance function:


\begin{equation*}{{O}_1} = - \mathop \sum \limits_{\left( {i,j} \right) \in E} {{w}_{ij}}\log {{p}_1}\left( {{{v}_i},{{v}_j}} \right),\end{equation*}

\begin{equation*}{{O}_2} = - \mathop \sum \limits_{\left( {i,j} \right) \in E} {{w}_{ij}}\log {{p}_2}\left( {{{v}_j}|{{v}_i}} \right),\end{equation*}

where *E* represents the edge set, *p*_1_ is the joint probability between vertex *v_i_* and *v_j_*, and *p*_2_ is the conditional distribution. The LINE package (https://github.com/tangjianpku/LINE) was used to reconstruct the input network with a parameter ‘-depth 2’. LINE models were trained for each of the orders with the default parameter ‘-size 100 -negative 5 -rho 0.025’, before model normalization and concatenation were performed. The dimensionality of the embedding vectors was reduced using the t-SNE algorithm, before performing *K*-means clustering with the t-SNE vectors.

### Bioinformatics analysis

All the publicly available datasets were converted into the hg38 coordinate using UCSC LiftOver (version 3.0). After removing the human housekeeping genes defined at HRT Atlas (28), edgeR (29) was used to detect the differentially expressed genes (DEGs), including differentially upregulated genes (DUGs) and differentially downregulated genes (DDGs), which satisfied the >2 FC and <0.05 false discovery rate (FDR) criteria. The FDR was estimated using the Benjamini–Hochberg correction. Two-tailed *t*-tests were used to estimate the significance of the ensemble of RCs. After applying Bonferroni correction, explanatory variables with an adjusted *P*-value <0.05 were considered to be statistically significant.

BEDTools (version 2.25) (30) was used to manipulate peaks, including intersecting and extending them within the genomic context. Cytoscape (version 3.9.1) (31) was employed to visualize the networks. Residual standard error (RSE) refers to the square root of an overall difference between TPM values and predictions, which was calculated by dividing the residual sum of squares by the degree of freedom (i.e. the total number of genes − 2). The RSEs gathered from multiple runs of regression modeling with different random seeds were averaged, and the mean RSE was used to measure the overall performance of models. The following packages in R language (https://www.r-project.org/) were used to perform the analyses and data visualization: ‘enrichGO’ in the ‘clusterProfiler’ package with a parameter ‘pAdjustMethod=BH, pvalueCutoff=0.01, qvalueCutoff=0.05’ for testing the enrichment of Gene Ontology (GO) biological process terms; ‘Rtsne’ with the default parameter along with principal component analysis for dimensionality reduction; ‘lm’ and ‘stepAIC’ for fitting regression models; and ‘hclust’ with the average-linkage method in Euclidean distance for hierarchical clustering.

## Results

### Overview of transcriptional regulation modeling

To profile the promoter-proximal and -distal regulators of specifically expressed genes, we initially explored the intrachromosomal interactions captured by Hi-C (6). We found that, in NB cells, 48.2% and 38.3% of the 133.2 million Hi-C contacts were located within the intergenic and gene body (GB) regions, respectively, and were paired with promoters in the regions 10 kb upstream of the TSS. Similarly, in GCB cells, 40.4% and 35.5% of the 54.5 million Hi-C contacts were promoter–intergenic and promoter–GB anchor pairs, respectively. By merging the 1-kb intergenic Hi-C anchors that overlapped with each other and were located at >10 kb from their core promoter (−2500 to +500 bp from the TSS), we were able to identify the paired anchors linking the intergenic regions to the core promoters. Thus, in NB cells, we identified 577 857 paired anchors contacting 7918 intergenic regions and 11 678 promoters, while in GCB cells, we identified 415 395 paired anchors contacting 7870 intergenic regions and 11 360 promoters.

**Figure 1.**
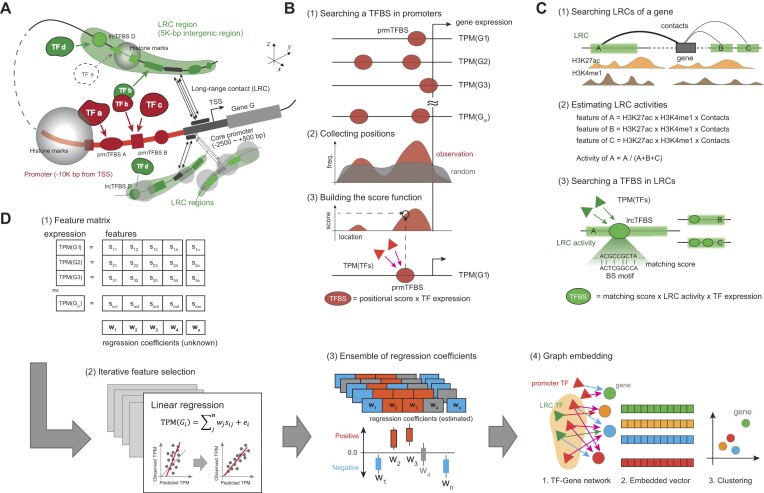
Overview of the computational pipeline to model gene expression regulation. (**A**) Schematic representation defining the 3D transcriptional domain for a gene to be modeled. (**B**) Scoring of proximal promoter TFBSs (prmTFBSs) by using positional information and the expression of TF genes. (**C**) Scoring of distal intergenic TFBSs (lrcTFBSs) based on the LRC activity and the expression of TF genes. (**D**) The analytical pipeline used to infer which regulators are important for gene expression using linear regression modeling and to identify coregulated genes using a graph-embedding method. LRC, long-range contact; TF, transcription factor; TFBS, TF binding site; TPM, transcripts per million; TSS, transcription start site.

Next, we increased the intergenic anchor window to within 5 kb from the center and defined them as LRC regions (Figure 1A). We supposed that these LRC regions include *cis*-regulatory elements forming the 3D transcriptional domain for a target gene. Lastly, we scanned the DNA sequences of promoters and LRC regions for identifying TFBSs and scored the TFBSs within the promoters (prmTFBSs) or LRC regions (lrcTFBSs). As described in previous studies (14,25), the scoring function for prmTFBSs takes into account the nonrandom positioning within promoters and the expression levels of the TF-coding genes whose products potentially bind to the prmTFBSs (Figure 1B). In addition, we scored the lrcTFBSs by estimating their LRC activities (Figure 1C). The lrcTFBS scoring method follows the principle in the activity-by-contact model (11), wherein an LRC region is weighted according to the Hi-C contact frequency, along with the extent of H3K27ac and H3K4me1 enrichment (see the ‘Materials and methods’ section for details). Collectively, we represented the 3D transcriptional domain of a gene by profiling the characteristics of prmTFBSs and lrcTFBSs (Supplementary Figure S1A–C).

To identify critical regulatory elements and determine how they interact in the gene regulatory network, by using the scored features of TFBSs as an input matrix, we built an analytical pipeline that integrated generalized linear regression modeling and graph embedding (Figure 1D). The pipeline followed a systematic approach, referred to as greedy iterative feature selection, to identify the explanatory features essential for predicting gene expression (14). The feature selection process was iteratively repeated to produce a collection of RCs (Supplementary Figure S1D–G). After performing statistical tests with these RC ensembles, we constructed TF–gene regulatory networks and mapped these networks to a low-dimensional vector space using the LINE graph-embedding method (15,27). This approach enabled clustering genes in the embedding space. The clustered genes reflect sharing a subregulatory network, i.e. a specific combination of TFs binding to the proximal and distal TFBSs. Furthermore, we extended the TF–gene networks by including cofactors annotated as known proteins physically interacting with the TFs (Supplementary Figure S1C). These networks represent tertiary regulatory complexes in the 3D transcriptional domain as an important factor in gene regulation, as recently reviewed (10,32,33).

### Genetic and epigenetic profiling in GC B-cell response

**Figure 2.**
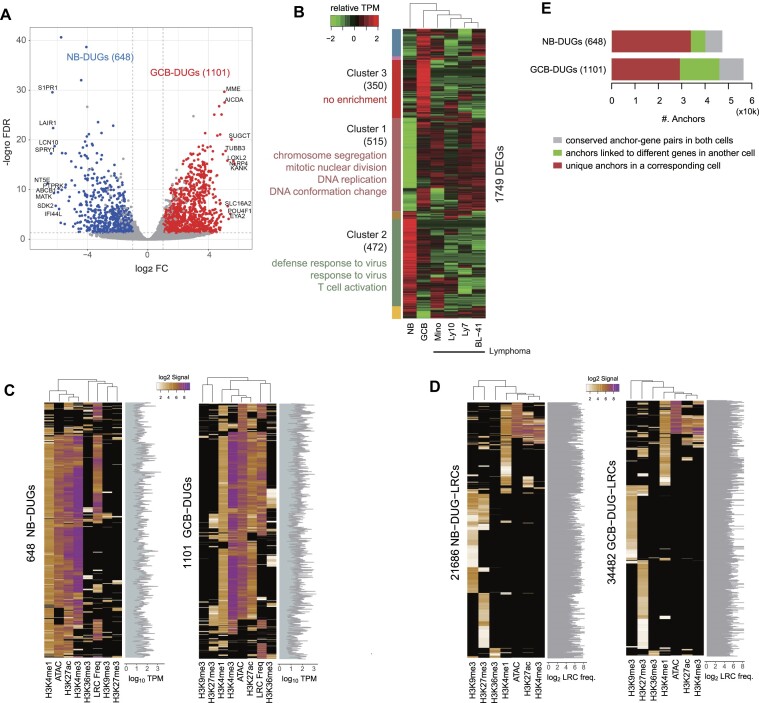
Genetic and epigenetic profiles in B-cell subtypes. (**A**) Volcano plot showing DEGs between NB and GCB cells. The top 10 genes in expression FC were text-labeled. (**B**) Expression profile of DEGs in healthy B cells and lymphoma cells. Clusters 1 and 2 were enriched with specific GO-BP terms (<0.05 *q*-value). (**C**) Genetic and epigenetic profiles of DEG promoters along with the expression TPM values. (**D**) Epigenetic profiles of LRC regions contacting NB-DUGs (NB-DUG-LRCs) and GCB-DUGs (GCB-DUG-LRCs). (**E**) Conserved or unique LRCs in the cell types. DEGs, differentially expressed genes; DUGs, differentially upregulated genes; FC, fold change; GO-BP, Gene Ontology biological process; FDR, false discovery rate; LRCs, long-range contacts; TPM, transcripts per million.

We identified 1749 DEGs that satisfied the >2 FC and <0.05 FDR criteria: 648 DUGs of NB cells (NB-DUGs) and 1101 DUGs of GCB cells (GCB-DUGs) (Figure 2A and Supplementary Table S2). By grouping the expression pattern of DEGs across healthy and lymphoma cells, we found seven clusters, two of which presented significant GO-term enrichments (Figure 2B): Cluster 1 was involved in cell proliferation as reported previously (34) and also observed in lymphoma. Clusters 2 and 3 were opposite patterns of each other. Unlike Cluster 2 involved in the immune responses, the DEGs in Cluster 3 were not enriched in any of the GO terms, suggesting that the genes are involved in broad biological processes potentially associated with GC B-cell reaction. We revisit this cluster later by investigating the coregulated gene clusters.

Next, we investigated the epigenetic profiles, which included six different histone modifications, chromatin accessibility and LRC frequency, around the core promoters of the DEGs (Figure 2C). The results indicated that these DEG promoters were mostly highly accessible and exhibited active histone signatures (i.e. H3K4me1, H3K4me3 and H3K27ac). However, none of these epigenetic signals clearly correlated with the gene expression levels (Figure 2C and Supplementary Figure S2A). Regarding the LRC regions, 34 260 out of 115 840 LRCs at NB cells (NB-LRCs) were linked to 381 NB-DUGs, 21 686 LRCs of which had either the histones or ATAC signals; 46 703 out of 71 472 LRCs at GCB cells (GCB-LRCs) were linked to 687 GCB-DUGs, 34 482 LRCs of which had the epigenetic signals (Figure 2D). Only 17% in the total NB-LRCs and 13% in the total GCB-LRCs were marked by H3K27ac and H3K4me1, enhancer-specific modifications. These signals were correlated with neither the Hi-C contact frequencies (Figure 2D) nor the targeted gene expression (Supplementary Figure S2B). Within these LRC regions, more than 80% of the constitutive Hi-C anchors were cell type specific (Figure 2E and Supplementary Table S3).

Collectively, our results support the notion that GC B-cell response is accompanied by the cell-type-specific reorganization of 3D genome structures and the activation of cell-cycle-associated pathways (35,36). Conversely, the specific subset of GCB-DUGs repressed in lymphoma was uncharacterized. Moreover, our profiles imply that the intricate interplay between multimodal genetic and epigenetic factors is required for tight gene regulation. These results suggest that further comprehensive analyses are necessary to understand the GC reaction.

### Identifying critical regulators by predicting gene expression

We searched TFBSs from the DNA sequences of promoters and LRC regions using the MATCH algorithm in the minimize false-positive mode and scored the TFBSs detected. Consequently, we identified 78 lrcTFBSs and 77 prmTFBSs among the NB-DEGs, as well as 79 lrcTFBSs and 78 prmTFBSs among the GCB-DEGs. Then, we used these TFBS features and histone signals as explanatory variables for predicting DEG expression (Figure 1D). To investigate the effects of LRC-mediated *cis*-regulatory elements, we built and tested eight regression models using different combinations of feature sets and target genes, in addition to the random shuffling of these features. A feature set containing only prmTFBSs was used as the B model. The B model was expanded by using a set of prmTFBSs and histone enrichment markers in the promoter region to generate the BH model. The BL model was constructed using a set of prmTFBSs and lrcTFBSs. Finally, the BHL model combined the features of the BH and BL models.

**Figure 3.**
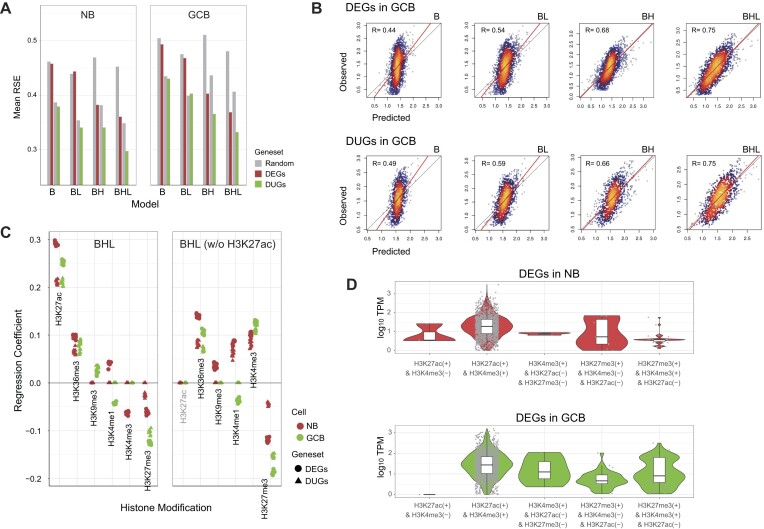
Performance of gene expression predictions and implication of histone modifications. (**A**) Performance of the four models (B, BH, BL, BHL) in predicting the expression of different gene sets measured by mean RSEs. (**B**) Scatter plots showing the correlations between the observed and predicted gene expression. (**C**) Distribution of the RC ensembles for the various histone marks with (left) and without (right) the inclusion of H3K27ac in the BHL model, which demonstrates the marked change in H3K4me3 RC. (**D**) Violin plots showing the combinatorial histone marks found on the DEG promoters, showing the dominant co-enrichment of H3K27ac with H3K4me3 and the bivalency of H3K27me3 and H3K4me3 for DEGs suppressed in NB versus GCB cells. B, the baseline model using prmTFBSs only; BH, the BH model using prmTFBSs and histone marks; BL, the BL model using prmTFBSs and lrcTFBSs; BHL, the BHL model combining the BH and BL models; *R*, Pearson’s correlation coefficient; RC, regression coefficient.

The BH model, which considers only the promoter configuration, significantly improved the predictive performance of the B and BL models (Figure 3A). Interestingly, the BHL model, combining the lrcTFBS features, further improved the identification of DUGs and DEGs in general (Figure 3B and Supplementary Figure S3A). These trends were also observed when predicting DUGs between K562 and THP-1 (Supplementary Table S4 and Supplementary Figure S4) and DUGs between K562 and OCI-LY7 (Supplementary Table S5 and Supplementary Figure S5) by using CRISPR-screened and predicted EPIs (Supplementary Tables S6–S8). On inspecting the selected features in each model, we found that the BHL model was based on the combinatorial effect of histone modifications and proximal/distal TFBSs (Supplementary Figure S3B). For example, in the prediction of GCB-DEG expression, 42 proximal/distal TFBSs were detected as having significant RCs (<0.05 adjusted *P*-value). The activity of a given TFBS was calculated by multiplying the mean RC by the total TPM value of binding TF genes. This assessment revealed whether a TFBS positively or negatively impacted the prediction of gene expression (Supplementary Figure S3C), indicating its potential role as an activator or a repressor, respectively.

In the BHL model, the explanatory features H3K27ac and H3K27me3 were estimated with remarkable RCs (Supplementary Figure S3B), which supports their importance in B-cell differentiation (37). In contrast, H3K4me3, an active promoter marker, was associated with negligible RCs or even negative RCs during the prediction of NB-DEGs (Figure 3C). To explain this observation, we next analyzed the distribution of histone markers in NB cells. We found that H3K27ac and H3K4me3 were frequently co-enriched during the expression of a wide range of genes (Figure 3D). This observation aligns with the transcriptional interplay previously reported between these two histone modifications (38,39). Of note, a subset of NB-DEGs, including those encoding cell adhesion molecules (e.g. protocadherin and LOXL2), were associated with the simultaneous presence of H3K4me3 and H3K27me3 but not H3K27ac, and their expression was particularly suppressed in NB cells. Therefore, it is reasonable to assume that the enrichment of H3K27ac was sufficient to explain the DEG expression, and the negative RCs of H3K4me3 likely accounted for the poised and repressed genes regulated by the bivalent chromatin domain (40,41). Indeed, removing H3K27ac from the model restored the significantly positive RCs of the H3K4me3 without sacrificing overall model performance (*R* = 0.74) (Figure 3C).

Taken together, as B and BH models improved the predictive performance when lrcTFBSs were added to the models, our results highlight that a synergy exists between the actions of promoter-proximal and -distal regulatory elements within the 3D transcriptional domain, which are also influenced by histone configuration in GC B-cell response (42). Moreover, our data suggest that H3K27ac is required for the accurate modeling of poised gene regulation in NB cells.

### Inferring the modes of proximal and distal regulatory effects

**Figure 4.**
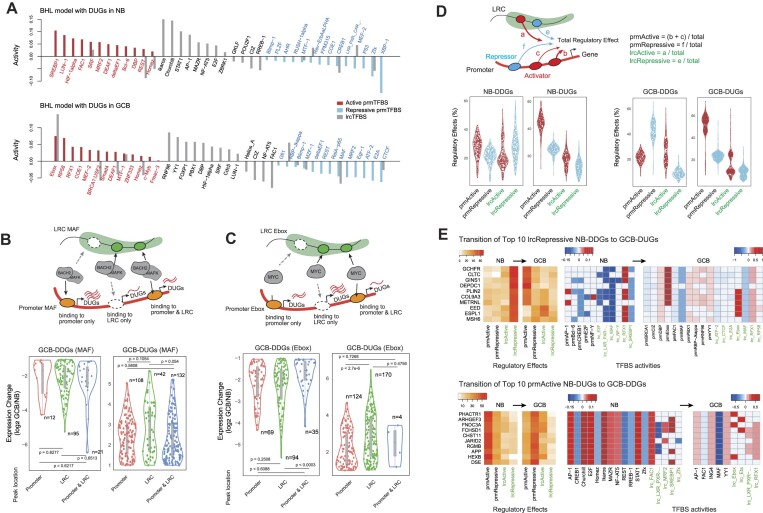
Contribution of proximal and distal regulatory effects inferred by BHL models. (**A**) Distribution of the activities of prmTFBSs and lrcTFBSs in the prediction of DUG expression (<0.05 adjusted *P*-value). (**B**) Schematic diagrams (upper) showing the category of the presence of BACH2/MAFK binding peaks. Violin plots (bottom) showing the gene expression changes in each category. (**C**) Schematic diagrams (upper) showing the category of the presence of MYC binding peaks. Violin plots (bottom) showing the gene expression changes in each category. (**D**) Schematic diagram (upper) and violin plots (bottom) showing the proportion of active and repressive effects exerted by prmTFBSs and lrcTFBSs for a target gene. (**E**) Examples of the transition of the regulatory effects for NB-DDGs to GCB-DUGs (upper) and for NB-DUGs to GCB-DDGs (bottom). TFBSs having >0.05 activities were shown. DDGs, differentially downregulated genes; DUGs, differentially upregulated genes; prm, promoter; lrc, long-range contact. Two-tailed *t*-test was used for calculating *P*-values.

Our regression models successfully identified well-documented transcriptional *cis*-regulators, such as Bcl-6, AP-1, Blimp-1, Bach2 and XBP1 (6,43,44), along with the diverse TFBS activities, according to their locations and target genes (Figure 4A). For instance, in the model that predicted NB-DUG expression using features in NB cells, Bcl-6 and Blimp-1 were identified in the promoter regions, while STAT1 and AP-1 originated from LRCs. Some TFBSs, such as REST, Homez and MEF-2, were detected in both the promoters and LRCs, where they exhibited mutually opposing activities. Although it has been reported that BCL6 binds promoter and intergenic regions in B cells (45), our modeling could not find statistical significance from most of its binding sites (Supplementary Table S9). This may be attributed to the systematic inference of the TFBS without using bona fide binding data.

To validate the inferred TFBS activities, we compared the publicly available peak positions of three TFs in GCB cells: *BACH2* and *MAFK* binding to the TFBS MAF (46), and *MYC* binding to the TFBS Ebox (47). Although the MAF was detected with negative RCs (Figure 4A), consistent with the repressive activities of *BACH2* and *MAFK* (43), most of the DDGs possessed the TF binding peaks at LRCs (Figure 4B). Interestingly, simultaneous peak presence at the promoters and LRCs was associated with gene suppression, even in the GCB-DUGs. For the *MYC* bindings, our model inferred the Ebox with positive RCs, consistent with its activating function (48). Unlike the MAF demonstrating the cooperative effect of promoter-bound and LRC-bound *BACH2* and *MAFK*, most of the *MYC* peaks were positioned at either the promoter or LRC regions (Figure 4C).

Next, we analyzed the TFBS activities by categorizing their locations and their modes of activation or repression (Figure 4D). Overall, both NB-DUGs and GCB-DUGs highly received positive regulatory effects originating from their promoters, which suggests the primary contribution of promoter-proximal regulators in gene upregulation. For example, as shown in Figure 4E, the top 10 NB-DDGs that most received the LRC-originated repressive effects at NB transit to GCB-DUGs strongly influenced by promoter-originated activators. Conversely, the top 10 NB-DUGs that most received the promoter-originated active effects at NB are repressed by mainly the promoter-proximal repressive effect at GCB, along with the combination of various active and repressive effects.

Collectively, these results show that 3D genome reorganization regulates the dynamic function of proximal and distal *cis*-regulatory elements in the transition of NB to GCB cells. Moreover, the prmTFBSs appear to be the main regulatory effectors, and the lrcTFBSs fine-tune transcription, consistent with a previous study (49).

### Characterizing 3D regulatory modules

**Figure 5.**
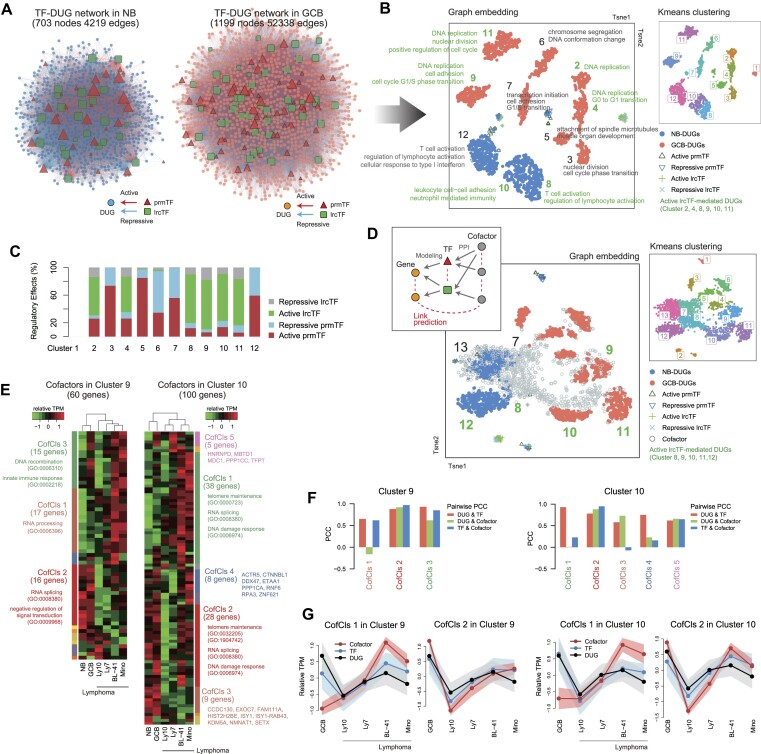
Results of the graph-embedding method with gene regulatory networks and the expression levels of clustered genes in lymphoma. (**A**) TF–gene regulatory networks inferred by the regression modeling. (**B**) Two-dimensional t-SNE plots depicting the embedded nodes of the TF–gene networks. The DEGs in each cluster share TF–gene connectivity, which implies coregulation, and are enriched in GO terms (<0.05 *q*-value), highlighted in green for lrcTF-involved clusters. (**C**) Proportion of activating and repressive effects exerted by the prmTFs and lrcTFs in each DEG cluster shown in panel (B). (**D**) Two-dimensional t-SNE plots depicting the embedded nodes of the cofactor–TF–gene networks. (**E**) Hierarchical clustering of cofactor expression in healthy B cells and lymphoma cells. The cofactors were derived from the corresponding clusters shown in panel (D). (**F**) Correlations among the mean expression pattern of cofactors, TFs and DUGs in GCB and lymphoma cells. PCC (Pearson’s correlation coefficient) measured the linear correlation of mean TPM values between cofactors and TFs linked together (blue), between the cofactors and DUGs linked by the TFs (green), and between the TFs and DUGs linked together (red). (**G**) Examples of correlated expression patterns of panel (F), which demonstrates the cofactor–TF modules that regulate the target GCB-DUGs. CofCls, cofactor cluster; TF, transcription factor; PPI, protein–protein interaction.

To explore how the 3D transcriptional domain combines the activities of promoter-proximal and -distal TFs, we constructed gene regulatory networks comprising TFs potentially binding to prmTFBSs and lrcTFBSs, named as prmTFs and lrcTFs, respectively. We identified 147 TFs with >3 TPM, of which 38% were DEGs in NB or GCB cells, comprising 82 prmTFs and 65 lrcTFs. Because multiple TFs can regulate a single gene, the resulting network was highly complex (Figure 5A). Thus, we employed a graph-embedding method (27) to convert the nodes (i.e. prmTFs, lrcTFs and DUGs) into 200-dimensional vectors based on the subgraph structures; these vectors were then clustered using a *K*-means algorithm as previously described (15). This approach clearly separated the nodes between the cell types and within a cell type, implying the presence of distinctive TF–gene connectivities in the regulation of NB-DUGs and GCB-DUGs (Figure 5B).

Next, we calculated TF activity scores by multiplying the mean RC of the TFBS by the TPM of the TF-coding gene. We found the markedly positive regulatory effects of lrcTFs in Clusters 2, 4, 8, 9, 10 and 11 (Figure 5C). Of note, although the 350 GCB-DUGs of Cluster 3, co-expressed genes shown in Figure 2B, were not enriched in any GO terms, these genes were scattered across all the GCB-related clusters (Supplementary Table S10) and enriched by crucial biological processes in Figure 5B. This result suggests that the models based on TF–gene regulatory connectivity are better at classifying functionally relevant genes than expression-based models. Interestingly, a specific GO term was repeatedly enriched in the different clusters as shown in Figure 5B. This result suggests that multiple sets of distinctive TF–gene interactions regulate the redundant biological processes in the transition of NB to GCB cells.

Given that several studies have reported the functional importance of cofactors in enhancer–promoter communication (50,51), we extended the TF–gene networks by adding 852 cofactor genes expressed in the cells (>3 TPM). These cofactors are annotated in STRING (26) as physically interacting with prmTFs and/or lrcTFs (>0.4 PPI score), and the cofactor-coding genes were non-DEGs. The cofactor–TF–gene networks included well-known interactions with coactivators (Supplementary Figure S6A), such as BRD4, EP300 and CREBBP (5,10,33,52). After embedding and clustering the nodes, we identified DUGs and co-clustered cofactors (Figure 5D and Supplementary Figure S7A) enriched by specific GO terms (Supplementary Figure S7B). For example, Clusters 8 and 12 comprised NB-DUGs, while Clusters 9, 10 and 11 included GCB-DUGs. These clusters were marked by LRC-originated regulatory effects and contained unique cofactors that interact with DUG-bound TFs, which differ from clusters of random networks (Supplementary Figure S6B). It is noteworthy that clustering with the cofactor–TF–gene interactions reduced the degree of cluster separation compared to that without cofactors (Supplementary Figure S6C). This is a natural result, as cofactors possibly interact with TFs associated with multiple gene clusters. Similar findings were observed when analyzing DUGs in K562, THP-1 and OCI-LY7 that might highlight cancer-type-specific cofactor–TF–gene interactions, and when comparing normal GCB cells and GCB-like lymphoma (Supplementary Figure S8).

Unexpectedly, the expression of cofactors was markedly altered in lymphoma versus normal B cells, and these changes were clustered (Figure 5E). Moreover, some of the CofCls correlated with the expression of their corresponding TFs and even DUGs targeted by these TFs (Supplementary Figure S7C and D). For example, the cofactor genes within CofCls 2 in Clusters 9 and 10 (Figure 5E), which were associated with RNA processing and the maintenance of chromosome structure, were expressed in conjunction with their directly interacting TF genes and DUGs targeted by these TFs (Figure 5F). This finding highlights the important role of cofactors, which are coregulated with proximal/distal TFs to form a 3D regulatory network. Interestingly, although CofCls 1 in Clusters 9 and 10 was associated with similar biological functions as CofCls 2 and repressed in normal B cells, the expression of cofactor genes in CofCls 1 exhibited correlations with the interacting TFs and DUGs in lymphoma cells (Figure 5G). Therefore, in line with the findings of a previous study (10), it is plausible that the dysregulation of cofactors disrupts cofactor–TF interactions in the 3D transcriptional domain and contributes to cancer development.

### Identifying the regulatory modules for BCL6

**Figure 6.**
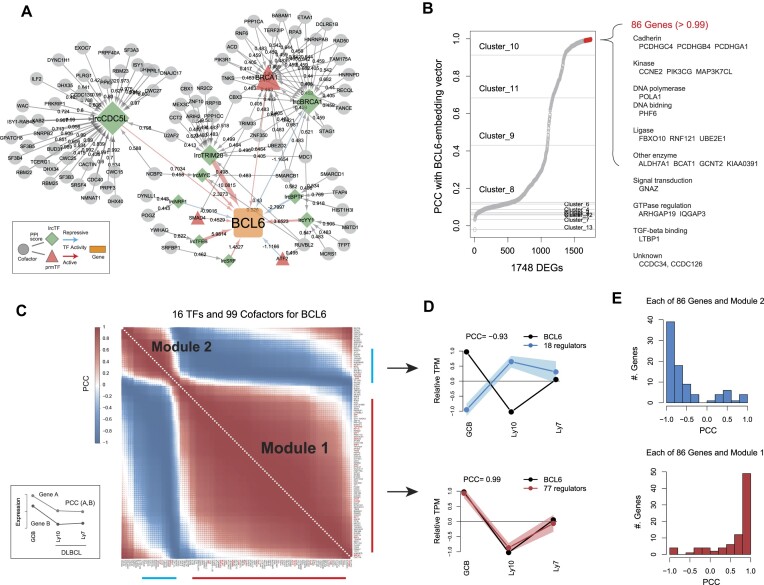
Cofactor–TF gene sets regulating BCL6 and presenting modular expression patterns in lymphoma. (**A**) The cofactor–TF connectivity network characterized Cluster 10 of Figure 5D and was inferred to regulate *BCL6*. (**B**) Distribution of correlation coefficients between the graph-embedding vector of *BCL6* and that of each of the DEGs, based on the graph structure presented in Supplementary Figure S6A. The cluster IDs correspond to those in Figure 5D. (**C**) Two regulatory modules of panel (A) showing similar expression pattern trends in GCB and lymphoma cells. (**D**) Correlation between the expression of *BCL6* and that of each of the modulated genes (>0.5 and <−0.5 PCCs) in GCB and DLBCL cells. (**E**) Distribution of gene expression correlation coefficients between *BCL6* and each of the 86 coregulated genes shown in panel (B) in GCB and DLBCL cells. DLBCL, diffuse large B-cell lymphoma; PCC, Pearson’s correlation coefficient.

To investigate the cofactor–TF modularity in more detail, we next focused specifically on *BCL6*, which is a master TF in GC B-cell response known to be controlled by LRCs (6,8). Overall, the regulatory network of *BCL6*, a GCB-DUG, comprised 13 prmTFs and 44 lrcTFs interacting with 681 cofactors (Supplementary Figure S9A). Among these regulators, the graph-embedding approach clustered *BCL6* and 99 cofactors, which belonged to Cluster 10 (Figure 5D) and interacted with 16 TFs (Figure 6A). To confirm the presence of *BCL6* neighboring genes in the embedding space, we calculated the PCCs for the embedded *BCL6* vector and that of each of the 1748 DEGs (Supplementary Table S11). As expected, we found that the higher PCCs were derived from the DEGs in Cluster 10 (Figure 6B). Specifically, we identified 86 GCB-DUGs that exhibited an exceptionally high degree of similarity in their embedded vector representations (PCC > 0.99). Since the similar embeddings mean having similar subnetwork structures, this result implies that the cofactors and TFs targeting *BCL6* (Figure 6A) coregulated these 86 GCB-DUGs, including cadherins and various enzymes.

The expression patterns of the 16 TFs and 99 cofactors (Figure 6A) in lymphoma cells frequently correlated with each other to form two regulatory modules (Figure 6C and Supplementary Figure S9B–D). In DLBCL, where *BCL6* serves as a therapeutic target (8), 77 regulators of the major module underwent a reduction in expression in a manner similar to *BCL6*; meanwhile, 18 regulators of the other module exhibited the opposite expression pattern (Figure 6D). These correlations were also observed for the 86 GCB-DUGs potentially coregulated with *BCL6* (Figure 6E). Collectively, our findings suggest that the cofactor–TF modules tightly regulate target genes as activators or repressors. Thus, the abnormal switching of those modules could potentially increase the risk of malignancy, particularly when accompanied by dysregulated chromatin reorganization due to change in the expression of architectural factors, such as *YY1*, *CTCF*, *STAG1*, *TRIM28*, *TFAP4* and *RAD50* (Supplementary Table S12).

## Discussion

To gain insights into the intricate connections between promoter-proximal and -distal transcriptional regulators, we modeled the interactions among cofactors, proximal/distal TFs and cell-type-specific genes, which were associated with spatial genome arrangement in GC B-cell response. We initially identified the TFBSs with the potential to bind TFs expressed in the 3D transcriptional domain, which were defined via cell-type-specific Hi-C contacts. Next, we profiled the TFBSs as nonredundant prmTFBSs and lrcTFBSs by scoring their characteristics. Our regression models subsequently used these data to recapitulate the gene expression profiles and successfully quantify the combinatorial regulation. Moreover, we observed highly complex regulatory networks from the interactions among DEGs and inferred TFs, as well as among TFs and their cofactors. We detected functionally relevant gene subsets from these networks by measuring the similarities among subnetwork structures: if two subnetworks of DEGs are similarly embedded in the vector space, the DEGs are supposed to be coregulated by similar regulatory connections of the TFs and cofactors.

We next identified the overarching trends through a comprehensive analysis of our computational modeling data. For instance, the predominantly activating effect of promoter-proximal elements upregulated 1749 DEGs in the B cells. The crucial function of distal elements, which further fine-tune transcription in a context-dependent manner, was subsequently exemplified by the MAF and Ebox. Notably, the spatial genome arrangement, associated with specific cell states (e.g. the quiescent NB cell state), was likely responsible for the distal repressive effects observed. In total, 1101 NB-DDGs were modeled in association with LRC-specific repressors in NB cells. These genes were upregulated during the transition of NB to GCB cells, in a process that was accompanied by the maintenance of pervasive H3K27ac-enriched chromatin, as well as a reduction in bivalent histone modifications and LRC-mediated repression. These findings underscore the importance of activators and repressors encoded in LRC regions (53), which are indispensable for precisely specifying cells by interacting with B-cell-lineage-specific epigenetic processes (2,54).

At the individual gene level, our results demonstrate that the complex 3D regulatory network, consisting of proximal/distal TFs and cofactors, exhibits specific subnetwork structures representing the node modularity, which is essential for regulating the expression of target genes. For example, *BCL6* expression in GCB cells was modeled using 58 TFs interacting with 681 cofactors, which formed a unique 3D regulatory module comprising 16 TFs interacting with 99 cofactors; this module was responsible for regulating a subset of GCB-DUGs. Remarkably, differentially coregulated DEG subsets exhibited similar functions, even though the specific connectivity characteristics of their 3D regulatory modules varied. This finding suggests that parallel pathways activated by distinct transcriptional protein complexes determine the level of cell-type-specific gene expression required for the regulation of B-cell identity. However, this functional redundancy is thought to increase the risk of cancer development (55). Indeed, we found that members of the 3D gene regulatory modules were frequently co-expressed with their target genes in lymphoma cells, although the causality of this relationship is still unclear. Nevertheless, understanding how changes in the 3D gene regulatory networks contribute to disease development will help the discovery of multiple biomarkers interacting with each other as modules.

## Conclusion

Our computational framework inferring the proximal and distal regulatory interactions by incorporating PPIs provides an alternative approach for deciphering transcriptional *cis*-regulatory code mediated by the 3D chromatin structures. It is noteworthy that our study is restricted by several difficulties. For example, due to relying on the DB annotations curated, we could not include factors, such as *IRF4*, *IRF8* and *FOXO1*, known to control GC B-cell response. In addition, the nature of data integration possibly causes artifacts in interpretation. Therefore, we further need to incorporate high-quality and large-scale muti-omics datasets for various cells and tissues and systematically investigate them. As a result, we will gain a deeper understanding of how the interplay between cell-specific *cis*-regulatory elements and the higher-order genome structure regulates gene expression.

## Supplementary Material

lqae050_Supplemental_Files

## Data Availability

All data used in this study were downloaded from public databases. The processed results are available as Supplementary data. The code is available on GitHub (https://github.com/Park-Sung-Joon/GLMGE) and Zenodo (https://doi.org/10.5281/zenodo.11046523).
