# Supplementary material for: A computational approach for deciphering the interactions between proximal and distal gene regulators in GC B-cell response
Source: NAR Genom Bioinform. 2024 May 6;6(2):lqae050. doi: 10.1093/nargab/lqae050 (PMC11071120; doi:10.1093/nargab/lqae050)
Supplement: lqae050_Supplemental_Files [file lqae050_supplemental_files.zip › Supplementary_Figures.pdf]

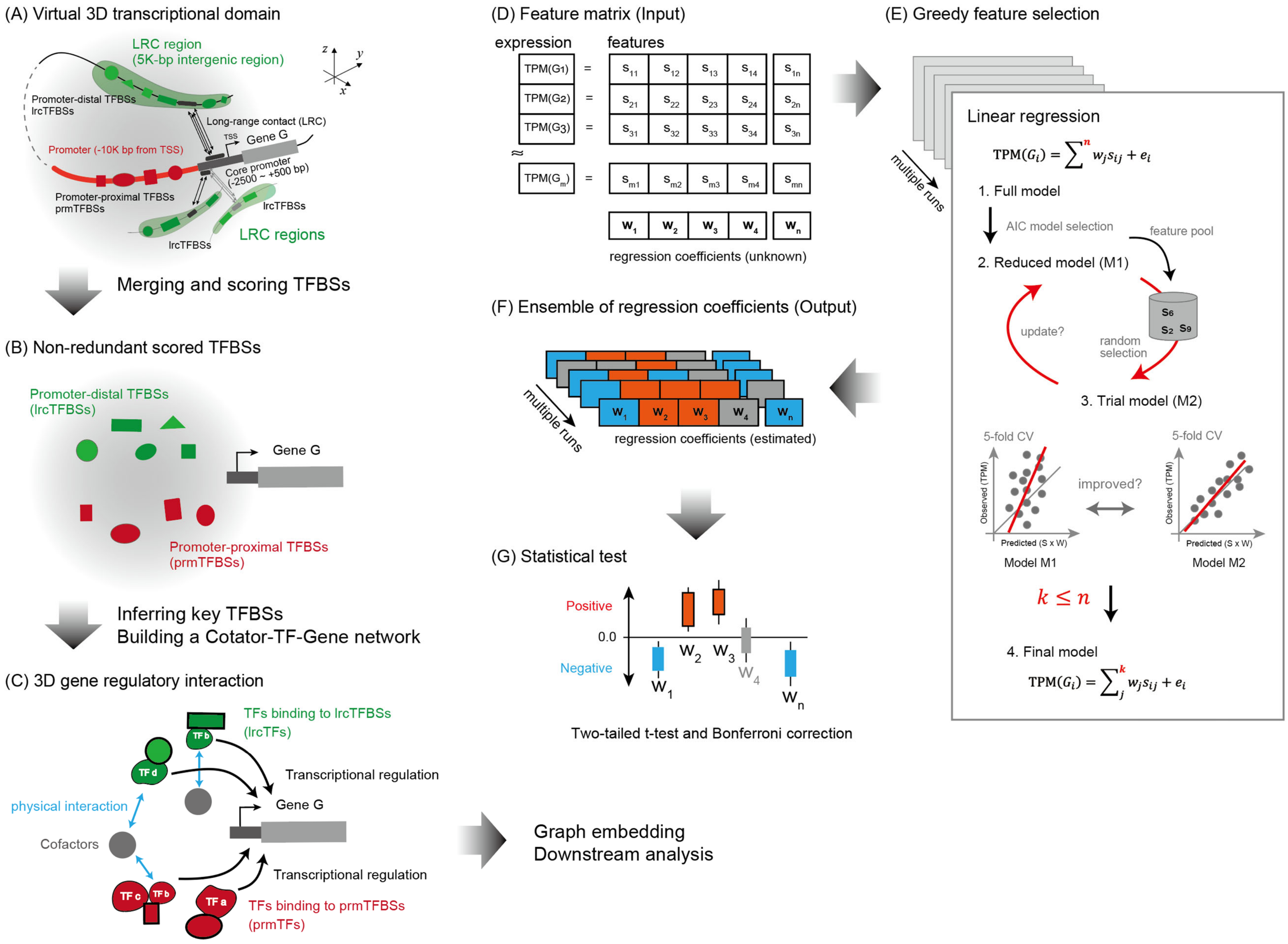

Figure S1: Schematic representation of modeling the 3D gene regulatory network. After defining the 3D transcriptional domain using Hi-C contacts, TFBSs are identified from the DNA sequences of promoters and LRC regions (A). The TFBSs are grouped into non-redundant prmTFBSs and lrcTFBSs and weighted by their genetic and epigenetic characteristics (B). After the linear regression modeling infers the key TFBSs, TFs potentially binding to the TFBSs and cofactors physically interacting with the TFs are prepared (C). Regarding detecting key TFBSs, starting with a full model of the input matrix that includes characteristics of regulatory elements (D), AIC reduces the model size, and 5-fold CV estimates the predictive performance (E). If the CV is much better than that of the current model, the current model is updated. This procedure is repeated with a variable removed by AIC. Also, this procedure runs several times with different random seeds. The final output is the regression coefficients for the input features (F) to be statistically tested (G). LRC, long-range contact; TF, transcription factor; TFBS, TF binding site; TPM, transcripts per million; CV, cross validation.

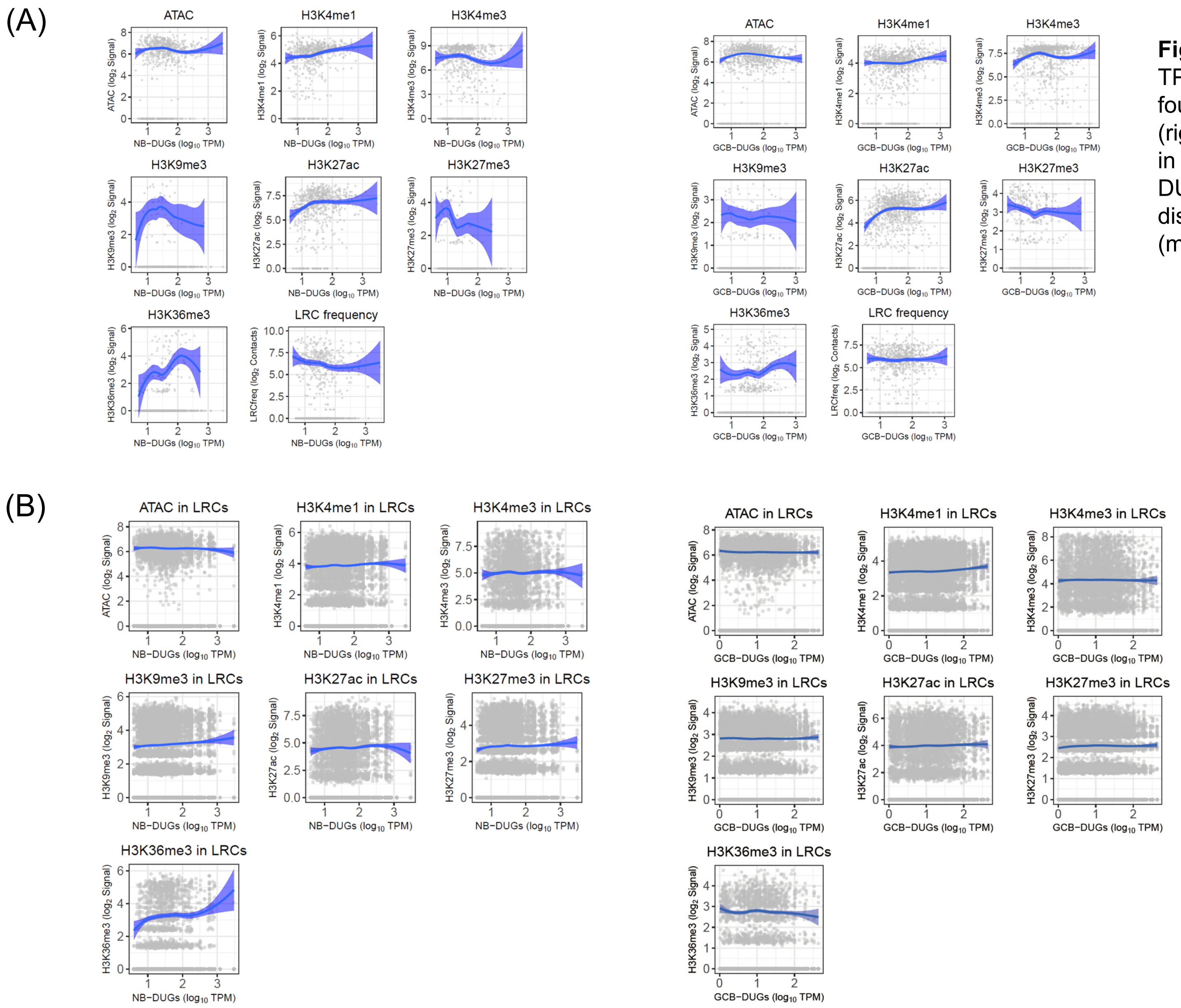

Figure S2: Distribution of epigenetic signals as a function of TPM found from promoters and LRC regions. (A) The signals found in the promoter regions of DUGs in NB (left) and GCB (right), which were used in Figure 2C. (B) The signals found in the LRC regions contacting NB-DUGs (left) and GCB-DUGs (right), which were used in Figure 2D. The blue line displays a smooth line depicted by geom_smooth (method="loess" and default parameters) in R.

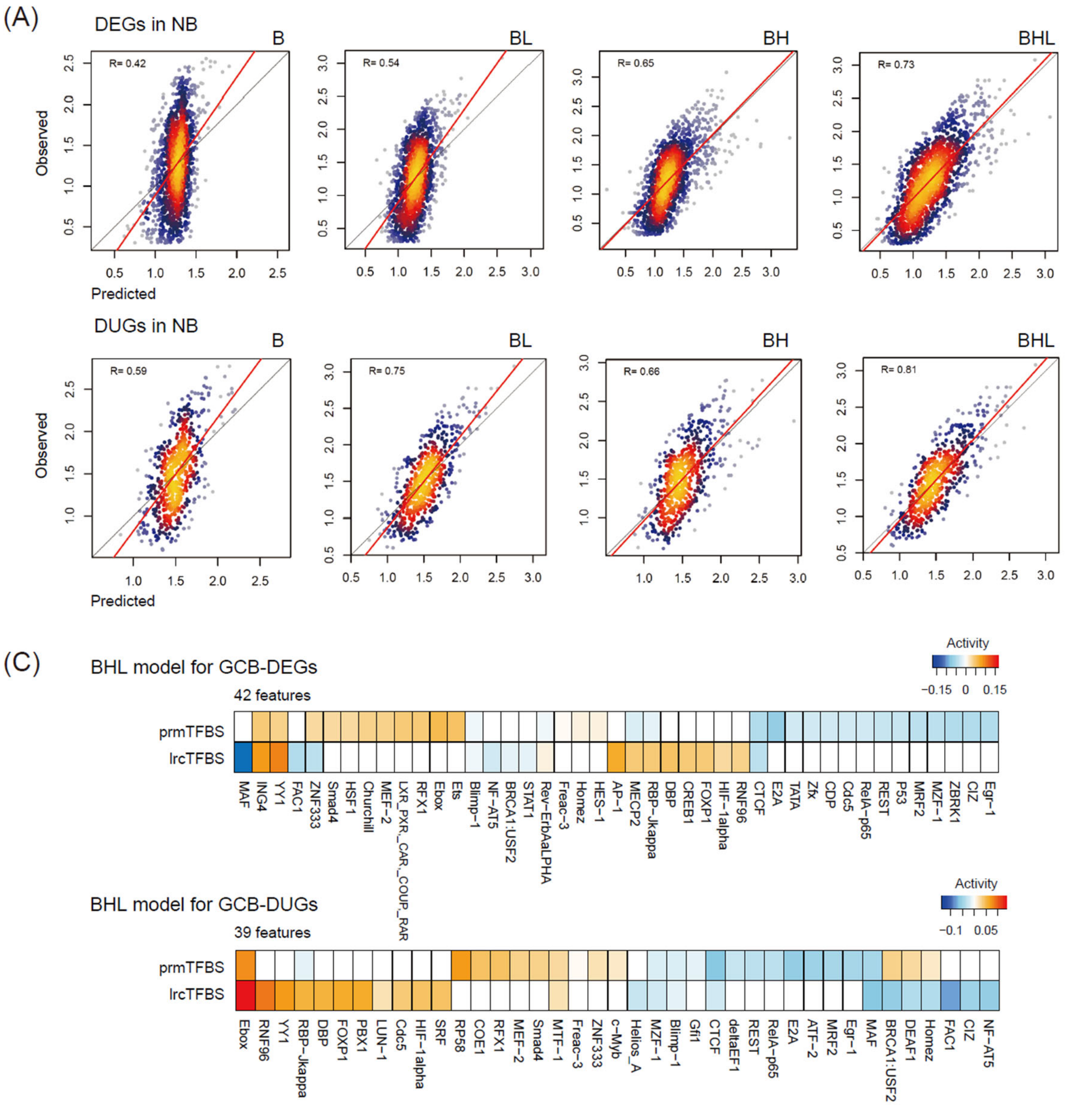

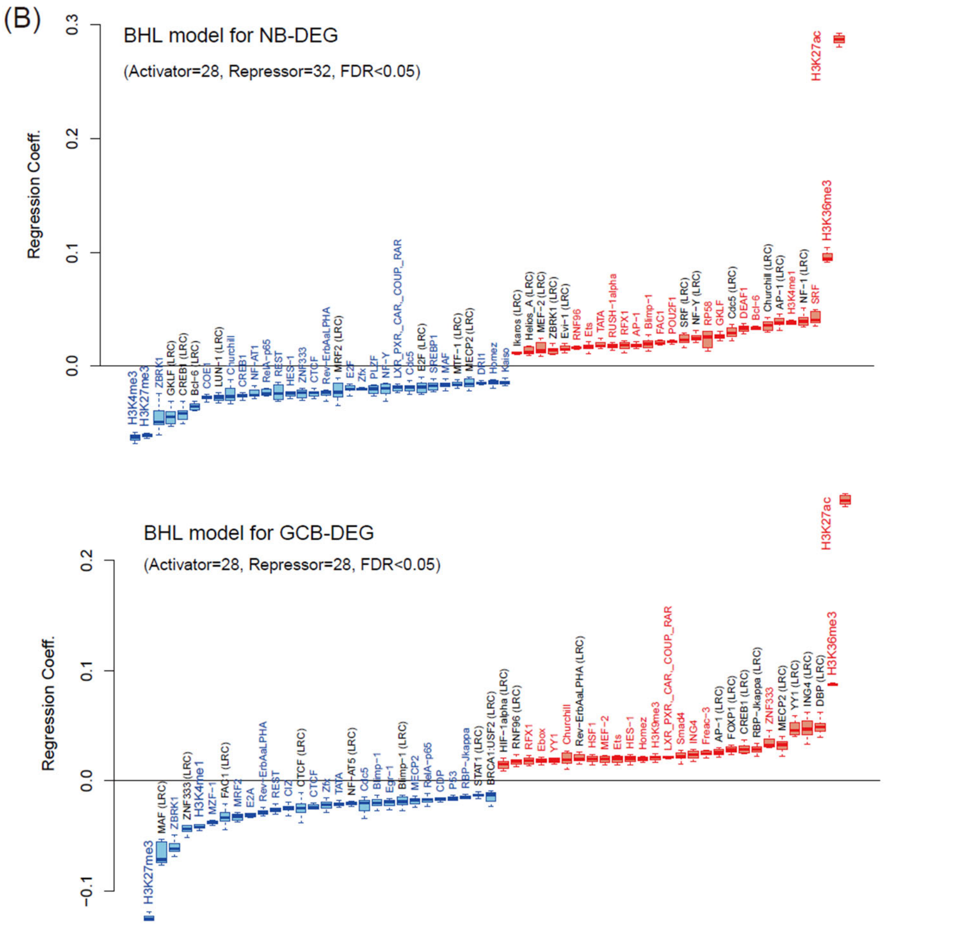

Figure S3: Predictive performance of the regression modeling of gene expression in NB cells and the distribution of the estimated RCs and TFBS activities. (A) Predictive performance of four regression models predicting gene expression in NB cells. (B) Distribution of estimated regression coefficients after filtering by FDR. (C) Distribution of TFBS activities in the BHL model of GCB cells.

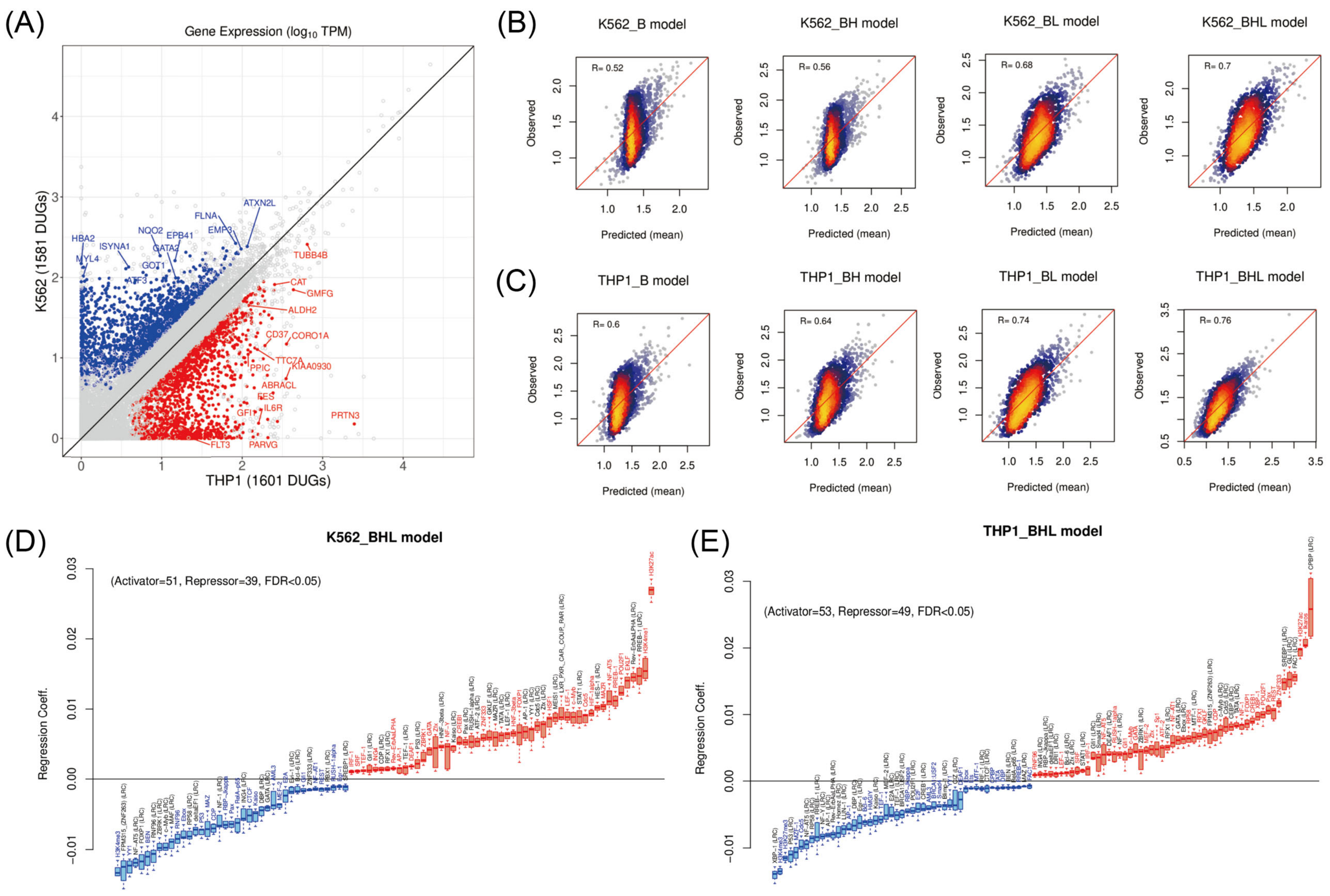

Figure S4: Predictive performance of the regression modeling for DUGs in the comparison of K562 and THP1. (A) Scatter plot showing DEG expression (>3 TPMs and > 2-fold changes). (B) and (C) Predictive performance of the four regression models. (D) and (E) Distribution of estimated regression coefficients.

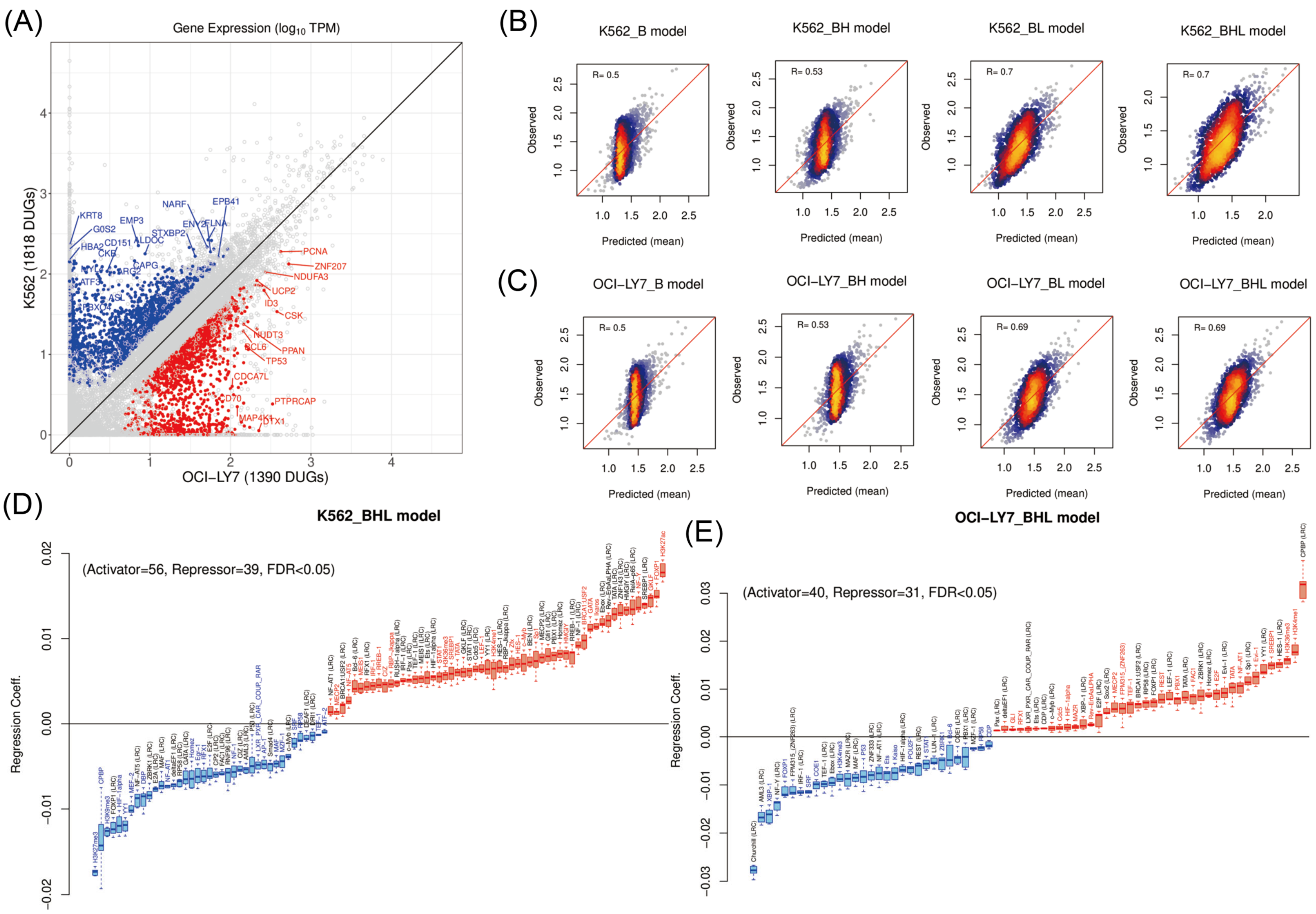

Figure S5: Predictive performance of the regression modeling for DUGs in the comparison of K562 and OCI-LY7. (A) Scatter plot showing DEG expression (>3 TPMs and > 2-fold changes). (B) and (C) Predictive performance of the four regression models. (D) and (E) Distribution of estimated regression coefficients.

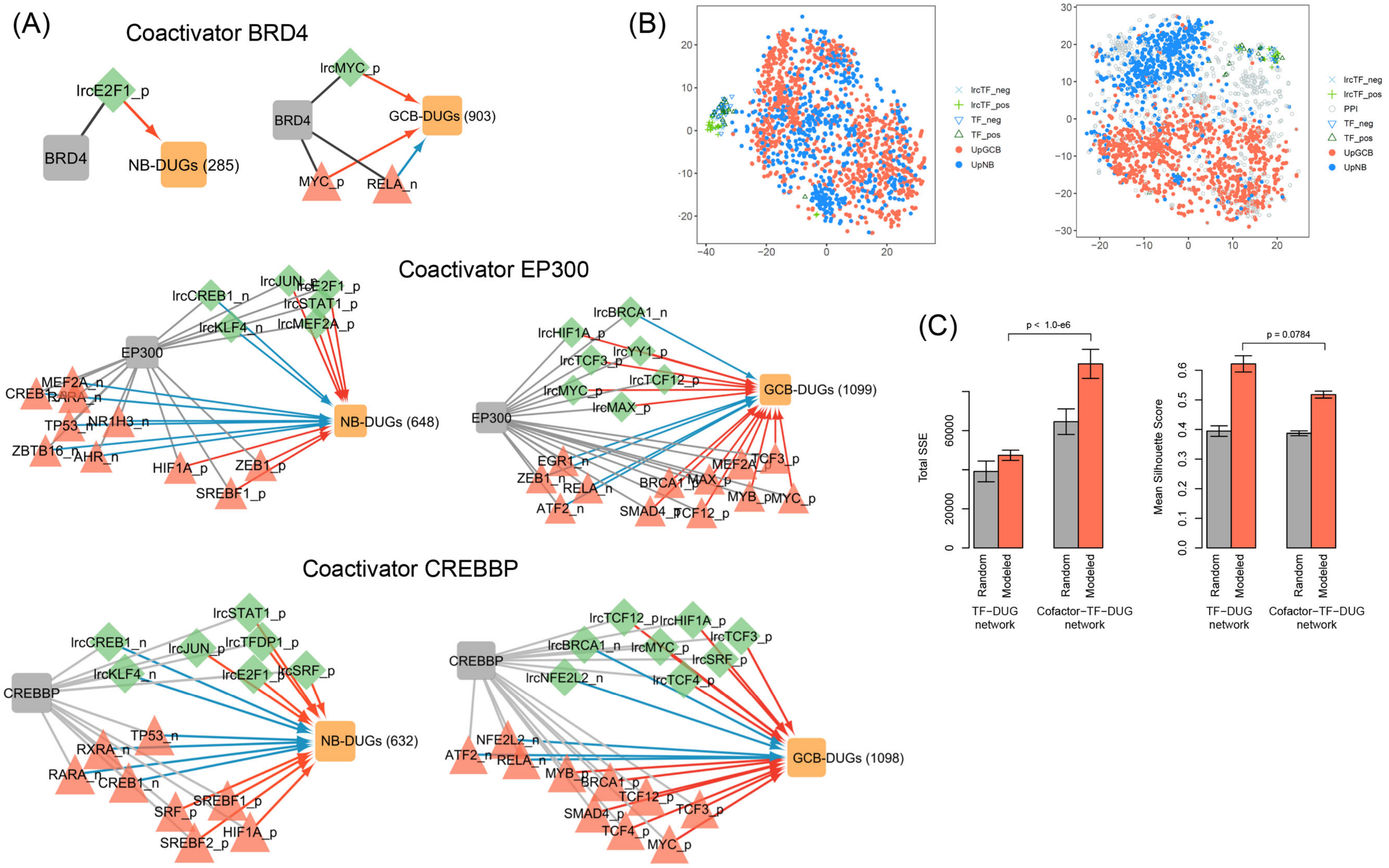

Figure S6: Examples of known coactivator interactions captured in this study and the clustering metrics of random and modelled networks. (A) Examples of well-known coactivators included in the Cofactor-TF-Gene networks of NB-DUGs and GCB-DUGs. (B) Examples of clustering of regulatory interactions with randomly shuffled edges. The TF-DUG network (left) is the one used in Figure 5B, and the Cofactor-TF-DUG network (right) is the one used in Figure 5D. The numbers of nodes and edges were retained, but the links were shuffled. (C) Cluster compactness and the degree of separation in clustering randomly generated and computationally modeled networks. SSE (sum of squared error) measures the compactness of each cluster (lower value is better), and the Silhouette score shows the degree of cluster separation where near +1 indicates that the clusters are far away from the neighboring clusters (higher value is better). Standard deviations were measured by running 10 times with different random seeds. “_p” and “_n” stand for “positive” and “negative”, respectively.

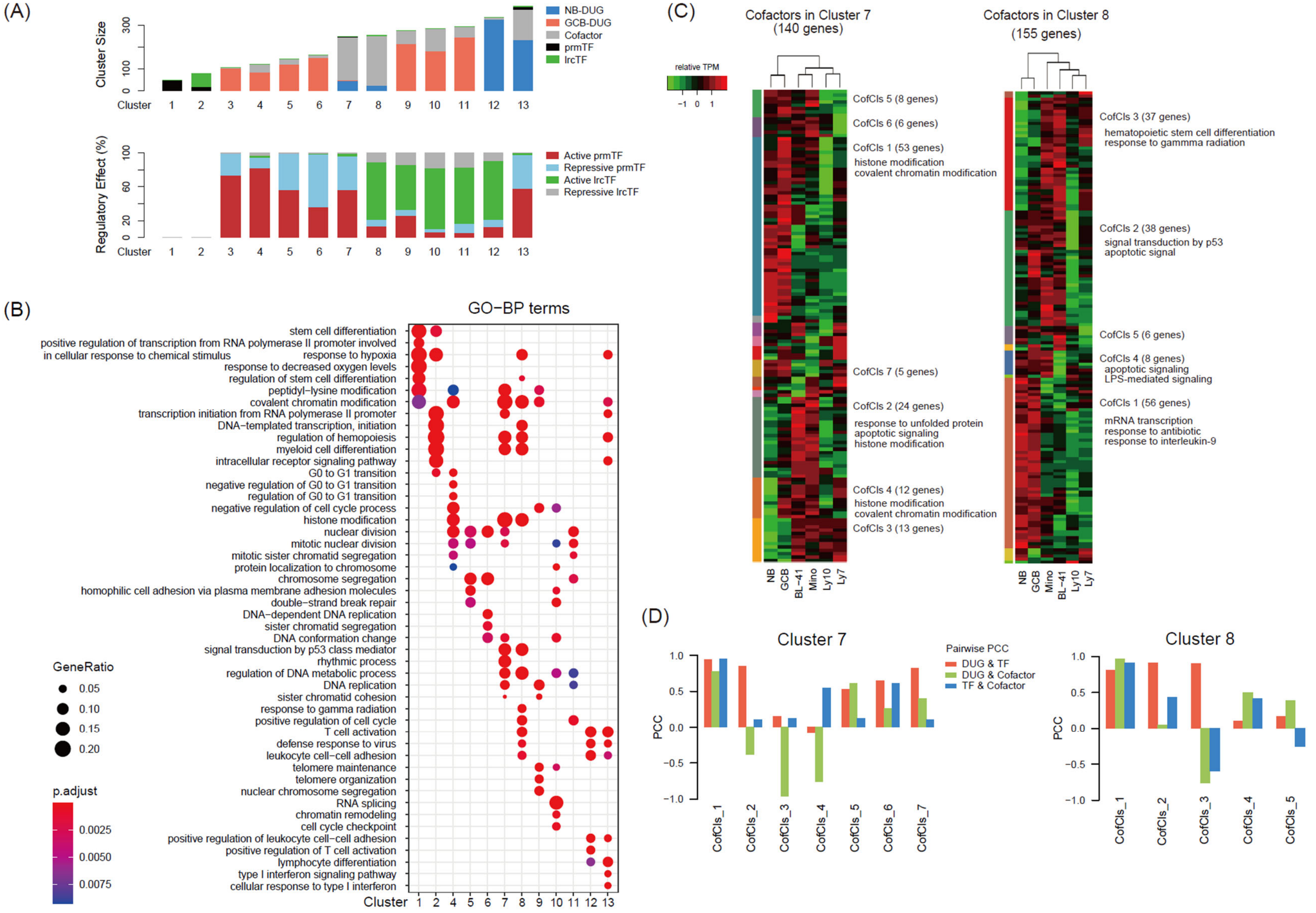

Figure S7: Further analyses of embedded clusters shown in Figure 5D. (A) The member composition of each embedded cluster, and the proportion of activating and repressive effects exerted by prmTFs and lrcTFs in each cluster. (B) The enrichment analysis of GO biological process terms (GO-BP terms) with the DEGs of each cluster. (C) Hierarchical clustering of the cofactor expression in healthy B cells and lymphoma cells, found from Clusters 7 and 8 in Figure 5D. (D) Correlations among the mean expression levels of Cofactors, TFs, and DUGs from Clusters 7 and 8 in GCB and lymphoma cells.

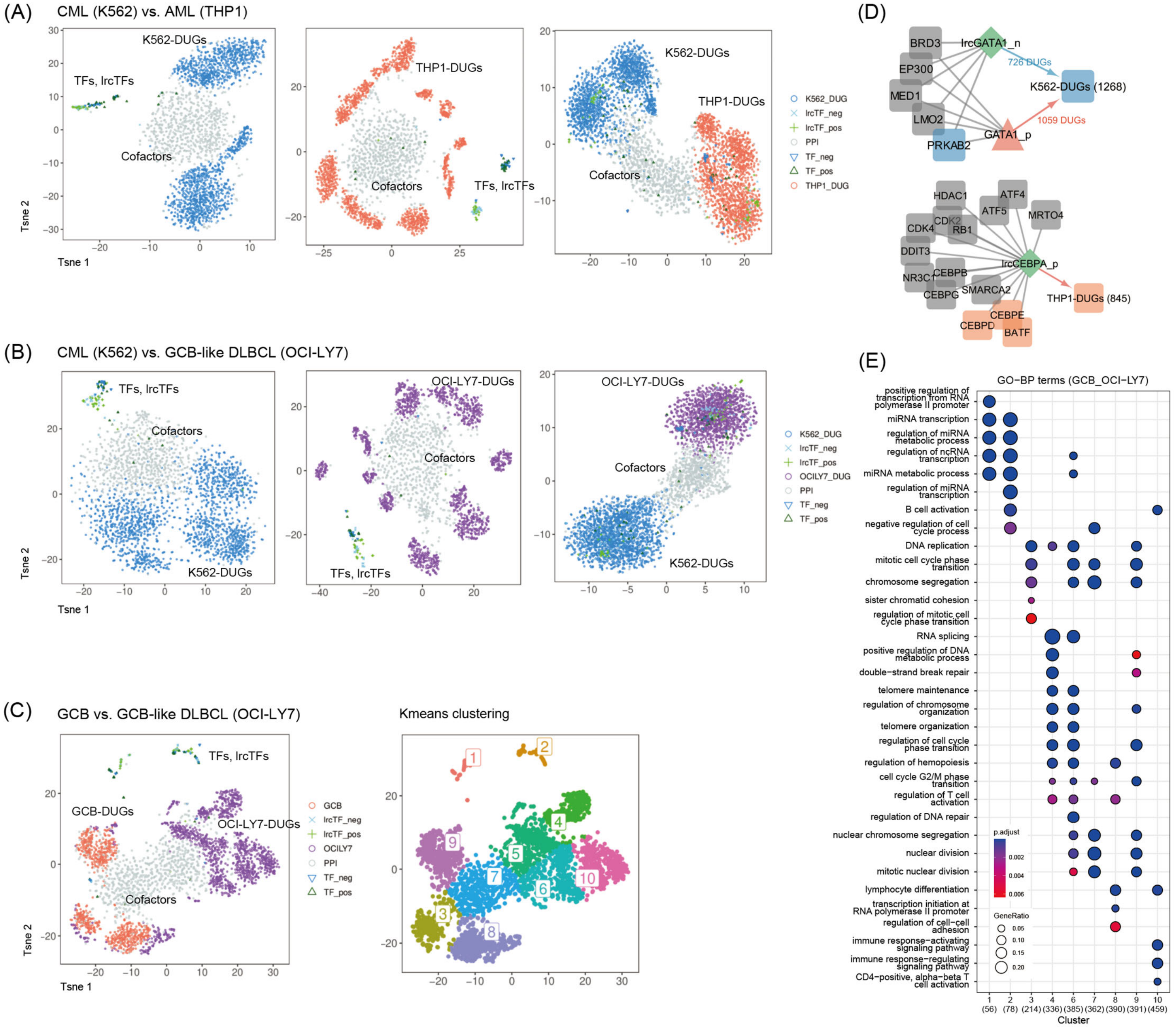

Figure S8: Graph-embedding and clustering of Cofactor-TF-gene interactions predicted from DUGs in K562, THP-1, and OCI-LY7, and in the comparison of GCB cells with GCB-like lymphoma. (A) Tsne plots showing the node clusters having similar Cofactor-TF-Gene interactions in K562-DUGs (left), THP1-DUGs (middle), and their aggregation (right). (B) Tsne plots showing the node clusters having similar Cofactor-TF-Gene interactions in K562-DUGs (left), OCI-LY7-DUGs (middle), and their aggregation (right). (C) Tsne plot showing graph-embedding and Kmeans clustering of Cofactor-TF-DUG interactions in comparing normal GCB with OCI-LY7. (D) Example of Cofactor-TF-gene sub-graphs showing well-known cancer-type-specify TF activities: GATA1 bindings in CML and CEBPA bindings in AML. (E) GO enrichment analysis with the Kmeans clusters shown in (C). Clusters 8 and 10 are of interest, including genes differentially regulated and involved in lymphocyte differentiation.

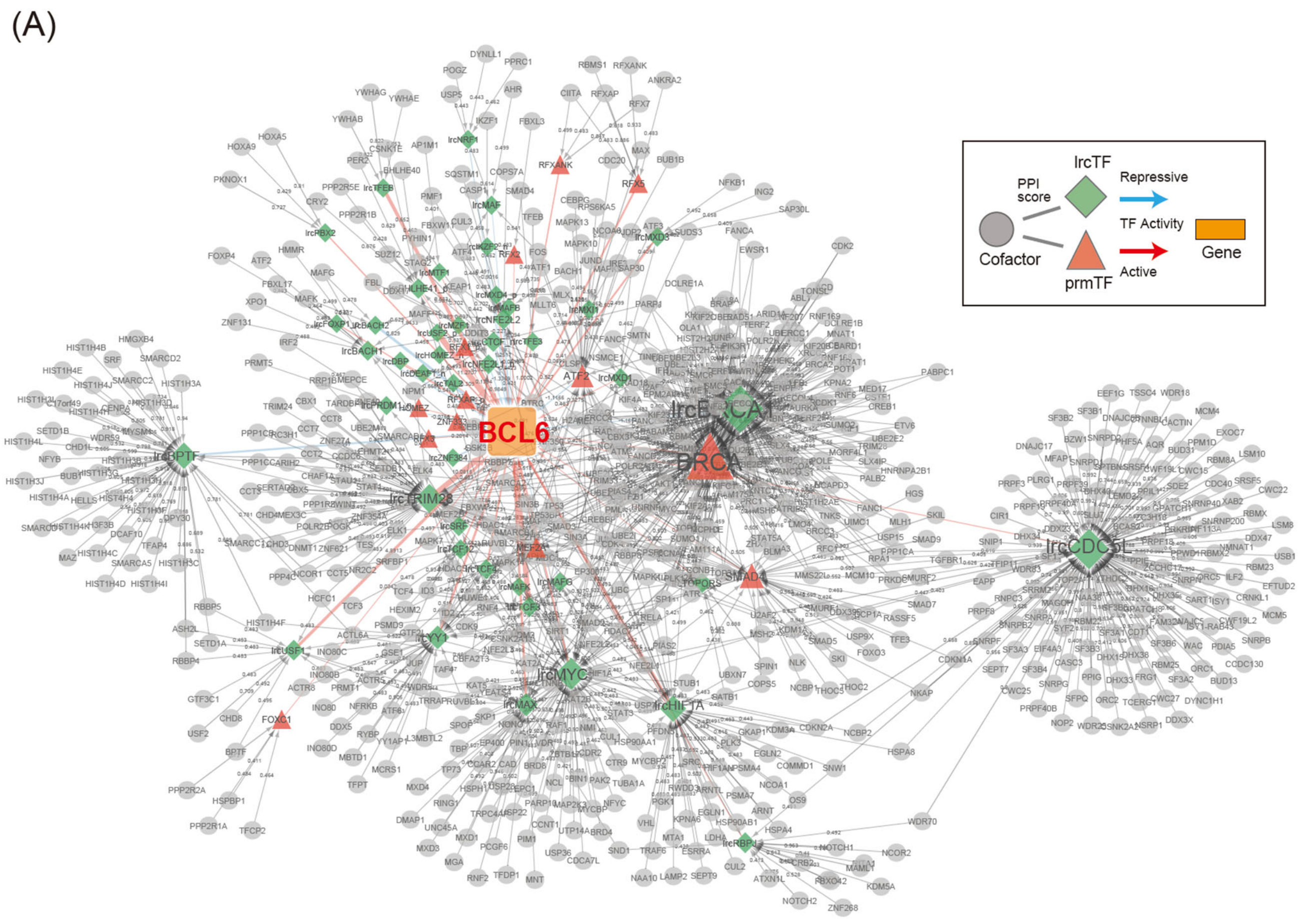

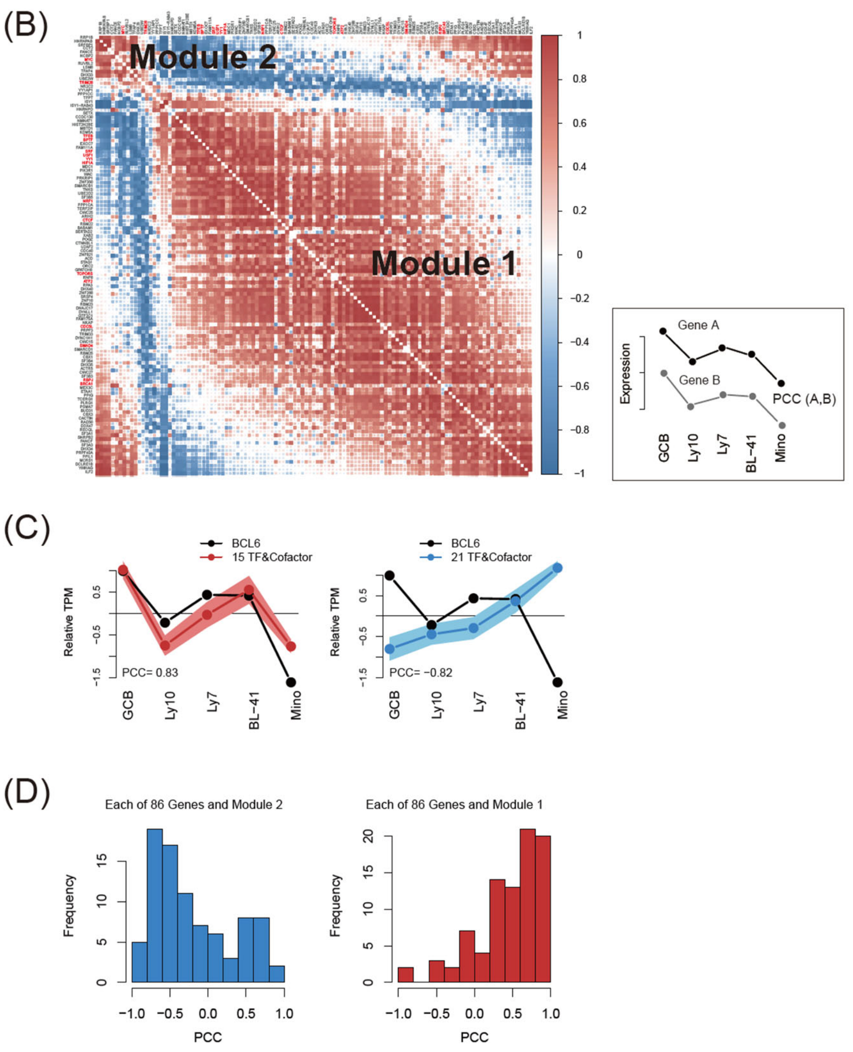

Figure S9: The Cofactor-TF-BCL6 network inferred from the regression model and the distribution of gene expression correlations of the BCL6 regulators in the cell lines. (A) The Cofactor-TF-BCL6 network inferred by the regression model. (B) The gene expression patterns of the two regulatory modules of Figure 6A in GCB and four lymphoma cells. (C) Correlation between the expression of BCL6 and that of genes in Module 1 (left) and in Module 2 (right). (D) Distribution of gene expression correlation coefficients between BCL6 and each of the 86 coregulated genes shown in Figure 6B in GCB and four lymphoma cells.
